# Estimation Accuracy on Execution Time of Run-Time Tasks in a Heterogeneous Distributed Environment

**DOI:** 10.3390/s16091386

**Published:** 2016-08-30

**Authors:** Qi Liu, Weidong Cai, Dandan Jin, Jian Shen, Zhangjie Fu, Xiaodong Liu, Nigel Linge

**Affiliations:** 1 Jiangsu Collaborative Innovation Centre of Atmospheric Environment and Equipment Technology (CICAEET), Nanjing University of Information Science & Technology, Nanjing 210044, China; qi.liu@nuist.edu.cn; 2 School of Computer & Software, Nanjing University of Information Science & Technology, Nanjing 210044, China; 18751971087@163.com; 3 Jiangsu Engineering Centre of Network Monitoring, Nanjing University of Information Science and Technology, Nanjing 210044, China; s_shenjian@126.com (J.S.); wwwfzj@126.com (Z.F.); 4 School of Computing, Edinburgh Napier University, 10 Colinton Road, Edinburgh EH10 5DT, UK; x.liu@napier.ac.uk; 5 Computer Networking and Telecommunications Research Centre, University of Salford, Salford, Greater Manchester M5 4WT, UK; n.linge@salford.ac.uk

**Keywords:** cloud computing, data convergence, MapReduce, data analysis, speculative execution, J0101

## Abstract

Distributed Computing has achieved tremendous development since cloud computing was proposed in 2006, and played a vital role promoting rapid growth of data collecting and analysis models, e.g., Internet of things, Cyber-Physical Systems, Big Data Analytics, etc. Hadoop has become a data convergence platform for sensor networks. As one of the core components, MapReduce facilitates allocating, processing and mining of collected large-scale data, where speculative execution strategies help solve straggler problems. However, there is still no efficient solution for accurate estimation on execution time of run-time tasks, which can affect task allocation and distribution in MapReduce. In this paper, task execution data have been collected and employed for the estimation. A two-phase regression (TPR) method is proposed to predict the finishing time of each task accurately. Detailed data of each task have drawn interests with detailed analysis report being made. According to the results, the prediction accuracy of concurrent tasks’ execution time can be improved, in particular for some regular jobs.

## 1. Introduction

In recent years, with the rapid development of social networking, internet of things, digital city and other new generation of large-scale network applications, smart data convergence technologies for faster processing big data generated by sensor networks or other devices are urgently required. To solve the above problem, in 2006 Google, Amazon and other companies proposed a “cloud computing” concept [1], presenting the use of network services anywhere and anytime on demand. It provides easy access to a shared resource pool (such as computing facilities, storage devices, applications, etc.). Through cloud computing, users can quickly apply or release resources according to their traffic load. Meanwhile, pay-as-you-consume cloud computing paradigm can improve the quality of services while reducing operation and maintenance costs [2].

Based on MapReduce (MR), Big Table and Google File System (GFS) proposed by Google, Hadoop has become a typical open-source cloud platform. Recently, it has been accepted and well used in both industry and academia due to its features of scalability, easy to deploy and high efficiency. Apart from Hadoop, some novel distributed platforms, e.g., Apache Storm [3] and Spark [4], have also been proposed and widely applied to process big data [5,6]. Apache Storm [3] is known as an efficient stream data preprocessing platform and has been steadily serving Twitter. Apache Spark [4] is another platform for big data processing. It is more applicable and has more capabilities, which consists of Spark Streaming [7], Spark SQL [8], MLlib [9] and GraphX [10]. All of the above modules make Spark a powerful platform. However, Hadoop is still a good choice for off-line computation, especially for a cluster lack of memory [11]. Actually, many research works based sensor networks have been continuously propelled. Several systems have been built based on Hadoop to speed up the procedure of sensor data analysis and data management [12,13,14,15,16].

As the core module of Hadoop, MR has been well investigated in order to improve the performance of job allocation and distribution. Scheduler is one of the critical parts in MR, which decides whether data can be processed efficiently. Previous work has been tremendously conducted on optimizing the Scheduler [17,18,19,20,21,22,23,24]. Apart from the scheduler, speculative execution strategies have also gained wide attention [25,26,27,28,29,30,31,32,33,34,35,36,37], since incorrect estimating the running duration of a run-time task may cause its inappropriate allocation. For periodically executed jobs, an optimized speculative execution strategy can effectively improve the performance of entire MR processing.

In this paper, a novel method is presented to improve the estimation accuracy of jobs’ execution time. Native Hadoop MapReduce is modified to collect data of run-time tasks. A linear model has been built based on the features of the data to predict the task finishing time more accurately.

The rest sections are organized as followed. Related work is introduced in Section 2, followed by Section 3, where our scheme for collecting historical data is presented. In Section 4, the relationship between progress and timestamp is analyzed and verified for predicting running time of run-time tasks, moreover, reasons for some phenomenon are discussed. Finally, conclusion and future work are presented in Section 5.

## 2. Related Work

Recently, with the development of cloud computing, many distributed big data progressing tools have been born. Hadoop, Storm [3] and Spark [4] that sponsored by Apache company have gained extensive attention due to their excellent performance. In stream processing field, Storm and Spark [3,4,5,6] have been the most important tools. Twitter used Storm to analyze and process data generated by social networks. Moreover, Alibaba, Baidu, Rocket Fuel, etc. also applied in their systems [3]. Except for Storm, Spark [4], as a new powerful framework, is being more and more attractive for some companies due to its convenience for machine learning and graph operator. Zaharia et al. proposed Spark Streaming [7], in which a novel recovery mechanism grants itself higher efficiency over traditional backup methods, and tolerance strategies [4]. Armbrust et al. presented a new module called Spark SQL [8]. Spark SQL provided rich DataFrame APIs and automatic optimization, which makes it significantly simpler and more efficient over previous systems. Spark MLlib offerd a wide range of functions for learning parameter settings, and APIs for a number of popular machine learning algorithms, including some potential statistics, linear regression, naive Bayes and support vector machines [9]. By taking advantage of distributed data flow architecture, GraphX brings low-cost fault tolerance and efficient graphic processing. On the basis of the data stream framework, GraphX achieved an exponential level performance optimization [10]. These frameworks usually have better performance while processing stream data. However, they have to pay for more memory consumption, which means that when it is applied in industry, companies have to pay more money. Furthermore, they do not have an obvious improvement over Hadoop in off-line and batch computation fields. Therefore, Hadoop is still a good choice, especially for a cluster lack of memory [11].

Many research works based sensor networks have been continuously propelled. Many systems have been built based to speed up the procedure of sensor data analysis [12,13,14,15,16]. Almeer speeded up remote sensing image analysis through an MR parallel platform [12]. Xu et al. introduced a Hadoop-based video transcoding system [13]. Hundreds of HD video streams in the wireless sensor networks can be parallelly transmitted due to the features of MR. Finally, better performance was achieved by optimizing some important configuration parameters. Jung et al. presented a distributed sensor node management system based on Hadoop MR [14]. With applying some specific MR and exploiting various crucial features of Hadoop, a dynamic sensor node management scheme was implemented. A solution for analyzing the sensory data was proposed in [15] based on Hadoop MR. According to the method, user behavior can be detected, and lifestyle trends can be accurately predicted. Alghussein et al. proposed a method based on MR to detect anomalous events by analyzing sensor data [16].

As the most critical part in MR, scheduler’s efficiency decides whether data can be processed efficiently and tremendous progress has been made now [17,18,19,20,21,22,23,24]. Apart from the scheduler, speculative execution strategies also gained wide attention with an increasing number of researchers concentrating on optimizing the performance of speculative execution [25,26,27,28,29,30,31,32,33].

A map task scheduling algorithm was presented to improve the overall performance of MapReduce calculations [17]. Their approach results in a more balanced distribution of the intermediate data. An approach to automating the construction of a job schedule was proposed that minimizes the completion time of such a set of MapReduce jobs [18]. Dynamic MR [19], which allows map and reduce slots to be allocated to each other, was proposed to facilitate the execution of the job. Yi proposed LsPS [20], a scheduler based on job size for higher efficiency of task assignments by abolishing the same response time. The execution efficiency of Memory-Intensive MapReduce applications was introduced in [21]. A resource-aware scheduler was proposed, in which a job is divided into phases. In each phase, a resource requirement is set constant, so phase-level scheduling can be achieved [22]. Saving resources of the cluster were conducted in [23], where a scheduler consisting of two algorithms called EMRSA-I and EMRSA-II was proposed. The disadvantages of current scheduler solutions for offline applications were analyzed in [24], and two algorithms were therefore presented, by which span and total finishing time can be decreased.

Recently, speculative execution strategies (SE) have been proposed [25,26,27,28,29,30,31,32,33,34,35,36,37]. Due to the unreasonable scheduling algorithms, SE strategy is used for solving the struggles and usually seen as a fault-tolerant mechanism. Proposed in Google, the speculative execution was implemented in Apache Hadoop and Microsoft Dryad. However, the current native SE strategy in Hadoop suffered from low accuracy [25]. Facebook disabled their SE strategy to avoid extra resource waste [26]. An optimized strategy, called LATE, was presented in [25], and weights of three stages (shuffle, sort, and reduce) in reduce task are set 1/3. MCP was proposed in [26], where data volume was considered when calculating the remaining time of run-time tasks. Maximizing Cost Performance was used as another limit for launching a backup task other than the difference between remaining time and backup time. EURL [27] was proposed where system load was seen as a key factor during calculating the remaining time of a task. An extended MCP was proposed while a load curve was added [28]. A smart strategy was proposed based on hardware performance and data volume of each phase [29]. In [30], the differences of work nodes were investigated to estimate backup tasks precisely. Optimal Time Algorithm (OTA) is another method that aims at improving the effectiveness of the strategy. However, the difference between the nodes’ processors are not well considered [31]. A new Speculative Execution algorithm based on C4.5 Decision Tree (SECDT) was proposed to predict execution time more accurately. In SECDT, completion time of straggling tasks is speculated based on the C5.4 decision tree [32]. Wang et al. proposed a strategy called Partial Speculative Execution (PSE) strategy. By leveraging the checkpoint of original tasks, the efficiency of the MR is therefore improved [33]. Adaptive Task Allocation Scheduler (ATAS) was presented to improve the original strategy. The ATAS reduces the response time and backs up tasks more quickly. Therefore, the success ratio of backup tasks is enhanced [34].

SE strategies based on the Microsoft’s distributed system have also been proposed. Using real-time progress reports, outliers of all tasks can be detected in an early stage of their lifetime [35]. Consequent actions setting free resources were then conducted to accelerate the overall job execution. To maximize job execution performance, Smart Clone Algorithm (SCA) was proposed in [36], which obtains workload thresholds used for speculative execution. The enhanced speculative execution (ESE) [37] algorithm was proposed for heavy clusters, as an extension of Microsoft Mantri programs.

Though many strategies have been proposed as above, detailed data of each task have drawn no interests with no detailed analysis report being made. Current existing SE strategies still have low accuracy while estimating the finishing time of running tasks.

## 3. A Two-Phase Regression Method

In this section, the method for gathering detailed information about each running task is introduced. Features of the data are then analyzed to facilitate prediction accuracy results for speculated tasks. Through the analysis of these data, the rule of the data tendency is discovered. Base on this, a new method called two-phase regression (TPR) is presented to predict the finishing time of running tasks more accurately, which is an optimized method of Linear Regression aiming at improving the accuracy.

### 3.1. Gathering Detailed Information of Each Running Task

**Figure 1:**
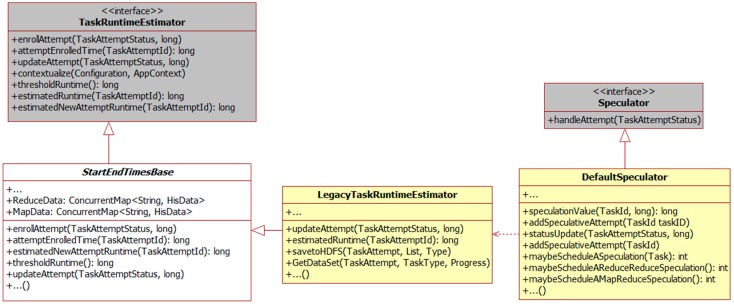
Implementation detail.

In Hadoop MapReduce, speculation is implemented in various classes. The relationship between these classes is shown in Figure 1. *TaskRuntimeEstimator* is an interface while *StartEndTimesBase* stores the lifetime of a complete task and it can be used for estimating the running time of a new task. The function *estimtedNewAttemptRunTime* is for calculating the finishing time of a backup task. Estimating time of a running task is calculated in a class called *LegacyTaskRuntimeEstimator*. The function *estimtedRunTime* is used for calculated the finishing time of the current task. The *updateAttmpt* function in both classes is a function for updating the task status every time when the progress updates. When a heartbeat arrives, *DefaultSpeculator* will start estimation processes to decide if a backup task needs to be created. The function *addSpeculativeAttempt* in it can be used for adding a backup task into a task pool. The *statusUpdate* updates the task status and call the functions to calculate the finishing time.

**Figure 2:**
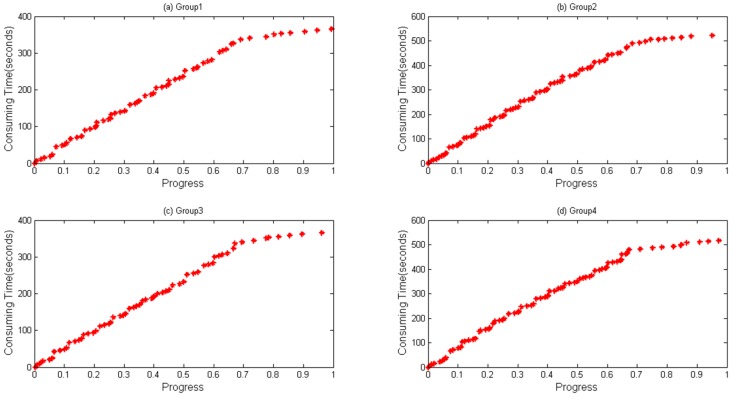
Data collected during running WordCount on the same node. Different groups of data generated from four map tasks running on the same node when executing WordCount: (**a**) Group 1; (**b**) Group 2; (**c**) Group 3; and (**d**) Group 4.

**Figure 3:**
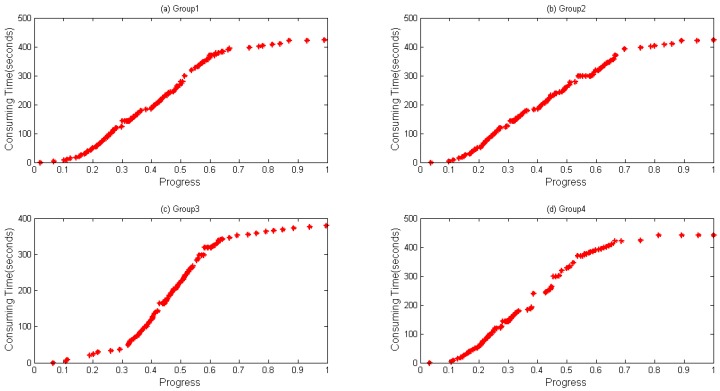
Data collected during running Sort on the same node. Different groups of data generated from four map tasks running on the same node when executing Sort: (**a**) Group 1; (**b**) Group 2; (**c**) Group 3; and (**d**) Group 4.

In Algorithm 1, a data structure called *HisPro* containing the chain (progress, timestamp) is used to store real-time information. When a task is to be completed, such a dataset will be generated and written to HDFS. α in Algorithm 1 is a threshold for evaluating if the dataset should be written to HDFS. It is an empirical value, and it is set 0.95 in this paper to ensure that the dataset will be stored before the task finishes. Time complexity equals the original time complexity due to the fact that data collecting method does not change the original logic procedure. Space complexity is O (*m* * *n*), where *m* is the task volume and *n* represents the data volume stored in each list. Figure 2 and Figure 3 show an example of collected data being generated when WordCount and Sort are executed, respectively. Through which, it can be seen that the same trend between progress and consumed time appears during the execution of WordCount and Sort.
**Algorithm 1:** Data Collecting Method (GetDataSet)**Input:***TA*: The task attempt*TN*: The name of the task attempt*P*: The progress of a running task*TT*: The type of the running task*DM*: The data map storing all the data lists of currently running tasks, whose key is *TN* and value is *DL**DL*: The data list storing *HisPro* generated by a running taskSteps:For each *TA* in the task pool  **If** Current *TA* is Running   Get the current HisPro   Get the *DL* from *DM* according to *TN*   **If**
*DL* does not contain *HisPro*    Add *HisPro* to the *DL*   **Else**    Update the *DL* using *HisPro*   **EndIf**   Update the *DL* in *DM*  **EndIf**  **If**
*P* > α   savetoHDFS (*TA*, *DL*, *TT*)  **EndIf****EndFor**

### 3.2. Data Analysis

In this section, a prediction algorithm, called Two-Phase Regression (TPR) algorithm is proposed, which can be divided into four steps: data preprocessing, data smoothing, data regression, and data prediction. Data storage structure is as (*p*, *Timestamp*).

**Step 1:** **Data Preprocessing.** The preprocessing function is shown in Equation (1), and final data structure can be expressed as (*p*, *t*). *p* represents progress and *t* is calculated according to Equation (1).
  (1) $$t_{i}=Timestamp_{i}-Timestamp_{min},$$
**Step 2: Data Smoothing.** Smoothing method is used for errors filtering, through which preprocessed data are smoothed with a five-point approximation. Specific smoothing processes are shown in Equation (2). Given a group of data, e.g., {6, 4, 5, 4, 3, 5}, then, the following values can be obtained: *T*(0) = 6, *T*(1) = 5, *T*(2) = 4.4, *T*(3) = 4.2, *T*(4) = 4, *T*(5) = 5.
**Step 3:** **Data Regression.** A mathematic model is shown in Equation (3), where a and b are two constants called regression parameters, while ε is a value near 0.

To work out the above function, Equation (4) is proposed as the first solve. $\hat{T}$, $\hat{a}$ and $\hat{b}$ are the estimation value of *T*, a and b, respectively

(2) $$\begin{array}{l} T(0)=t(0)\\ T(1)=(t(0)+t(1)+t(2))/3\\ T(2)=(t(0)+t(1)+t(2)+t(3)+t(4)+t(5))/5\\ T(3)=(t(0)+t(1)+t(2)+t(3)+t(4)+t(5)+t(6)+t(7))/7\\ \ldots \\ T(n-2)=(t(n-4)+t(n-3)+t(n-2)+t(n-1)+t(n))/5\\ T(n-1)=(t(n-2)+t(n-1)+t(n))/3\\ T(n)=t(n) \end{array},$$

(3) $$\{\begin{array}{l} T=ap+b+\varepsilon \\ \varepsilon \sim N(0,\sigma^{2}) \end{array}\;\left(T\sim N(ap+b,\sigma^{2})\right)$$

(4) $$\hat{T}=\hat{a}p+\hat{b}$$

Then, (*p*, *t*) can be substituted into Equation (1), as expressed in Equation (5).

(5) $$\begin{array}{cc} T_{i}=ap_{i}+b+\varepsilon_{i} & \left(i=1,2,\ldots ,n\right) \end{array}$$

Least Squares [38] is used to calculate the minimum value of the function Q(a,b), as described in Equation (6), for the purpose of improving the accuracy of the method.

(6) $$\underset{i}{min}\;Q(a,b)=\sum_{i=1}^{n}{\left[T_{i}-(ap_{i}+b)\right]}^{2}$$

Equations (7) and (8) are apartial differential equations generated by Equation (6), in which, $T_{i}$ and $p_{i}$ in these two equations are seen as constants, a and b are variables. A Solutions to Equations (7) and (8) are recorded as $\hat{a}$ and $\hat{b}$, which are the least square estimation of a and b, respectively

(7) $$\frac{\partial Q(a,b)}{\partial b}=-2\sum_{i=1}^{n}\left(T_{i}-ap_{i}-b\right)=0$$

(8) $$\frac{\partial Q(a,b)}{\partial a}=-2\sum_{i=1}^{n}\left(T_{i}-ap_{i}-b\right)p_{i}=0$$

Finally, $\hat{a}$ and $\hat{b}$ can be calculated according to Equations (9) and (10), respectively, where $\hat{a}$ is the slope of point ($p_{i}$, $T_{i}$), so we can calculate it according to Equation (10), whereas $\bar{T}$and $\bar{p}$ are obtained by Equation (11). $\hat{T}=\hat{a}p+\hat{b}$ can be seen as the regression function of Equation (12).

(9) $$\hat{b}=\bar{T}-\hat{a}\bar{p}$$

(10) $$\hat{a}=\frac{l_{pt}}{l_{pp}}=\frac{\sum_{i}\left(p_{i}-\bar{p})(T_{i}-\bar{T}\right)}{\sum_{i}{\left(p-\bar{p}\right)}^{2}}$$

(11) $$\bar{p}=\frac{1}{n}\sum_{i}^{n}p_{i},\bar{T}=\frac{1}{n}\sum_{i}^{n}T_{i}$$

(12) $$t=E(T)=f(p_{1},p_{2},\ldots ,p_{n})$$

The TPR can be seen as an extension of part (1) of Equation (13), where λ is a threshold set as 0.67, decided by the point where the slope has the biggest change. When p equals λ, an equation shown in Equation (14) can be obtained. According to the continuity of the data tendency, the value of part (1) and part (2) in Equation (14) are equivalent. Thus, d can be described as Equation (15). Finally, Equation (16) can be obtained.

(13) $$\{\begin{array}{l} T=ap+b+\varepsilon \\ T=c(p-\lambda )+d+\varepsilon \end{array}\begin{array}{c} p<=\lambda \\ p>\lambda \end{array}\begin{array}{c} \left(1\right)\\ \left(2\right) \end{array},$$

(14) $$\{\begin{array}{ll} T=a\lambda +b+\varepsilon & \left(1\right)\\ T=d+\varepsilon & \left(2\right) \end{array},$$

(15) d=aλ+b

(16) $$\{\begin{array}{l} T=ap+b+\varepsilon \\ T=cp+(a-c)\lambda +b+\varepsilon \end{array}\begin{array}{c} p<=\lambda \\ p>\lambda \end{array}\begin{array}{c} \left(1\right)\\ \left(2\right) \end{array},$$

**Step 4: Data Prediction.** Data are firstly divided into two equal parts, A and B. Top 1/3 of part A is dropped to avoid potential errors created by the initial phase. Then, Part A is used for training phase to find parameters (a, b). The variable (c) in Equation (16) is calculated according to the mean value of data generated in the same node. Part B is used for validation.

## 4. Experiments and Results

### 4.1. Data Collection and Its Results

In order to establish a practical performance testing environment, a Hadoop cluster consisting of eight virtual machines has been set up in a server. The server is equipped with four Intel^®^ Xeon^®^ CPU E5649 2.53 GHz six-core-processors, 10 TB hard drive and 288 GB memory. The speed of hard drives is about 144 MB/s. Those virtual machines are connected to a 1000 Mbps switch according to bridge mode. The specification of each machine is shown in Table 1, and the version of Hadoop is 2.6.0. Table 2 shows input data volume of Sort and WordCount, as well as data volume of map tasks collected by our gathering module through running jobs for multi times. Data sets in [39] are used as for testing scenarios. These data sets are freely provided by Purdue MapReduce Benchmarks Suite. The Benchmarks Suite has been widely used for evaluating MR optimization.

Result files are stored in the local HDFS as shown in Table 3. All Files are named with “attempt”, TimeStamp, JobId, “m”, TaskId, attemptId and “MAP”, separated by “_”, e.g., “attempt_1460088439095_0005_m_000008_0_ MAP”. Table 4 shows the data structure of a task file, which contains two columns, i.e., progress and timestamp, respectively.

### 4.2. Data Analysis and Prediction

**Figure 4:**
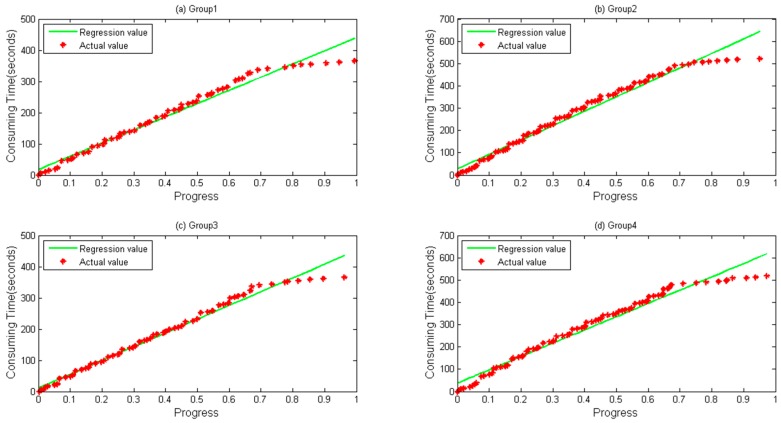
Linear Regression calculated by direct regression algorithm during a lifetime. (**a**) Group 1; (**b**) Group 2; (**c**) Group 3; and (**d**) Group 4 data and their values calculated by Linear Regression for WordCount sample.

**Figure 5:**
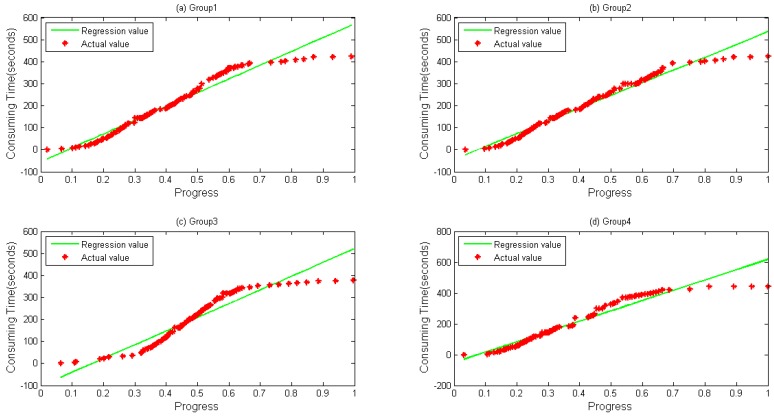
Linear Regression calculated by direct regression algorithm during a lifetime. (**a**) Group 1; (**b**) Group 2; (**c**) Group 3; and (**d**) Group 4 data and their values calculated by Linear Regression for Sort sample.

A notebook configured with 12 GB memory, Core^®^ i3 dual-core-processors and a 500 GB hard disk is used for parameter optimization and prediction time. Data files are first copied to localhost from HDFS. RMSE and MAPE, described in Equations (17) and (18), respectively, are selected as our evaluation indicators, where T′ is the predicted value and T represents the actual value when the progress reaches i. Figure 4 and Figure 5 depict four groups of data fitness for WordCount and Sort during their whole lifetime when the regression function is applied. In Figure 4, regression values fit actual values when the progress is below about 0.65, but does not perform very well when a task is going to be accomplished, during which differences between them gradually happen.

Figure 5 shows the data fitness of sort data, where a similar tendency happens as in Figure 4 with a bit more differences when a task is at its very beginning.

(17) $$RMSE=\sqrt{\sum_{i=1}^{n}\frac{{\left({T_{i}}^{\prime }-T_{i}\right)}^{2}}{n}},$$

(18) $$MAPE=\frac{\sum_{i=1}^{n}\left(|{T_{i}}^{\prime }-T_{i}\right|/T_{i})}{n},$$

The TPR method is then implemented, as shown in Table 5 and Table 6. Twenty-four groups of data are randomly selected and evaluated using the MRSE and MAPE. For WordCount sample shown in Table 5, most MRSE values are less than 2 and MAPE values are under 5%. A similar tendency can be found in Sort Sample shown in Table 6. In Table 5, the average RMSE value of WordCount is 1.7, and the average MAPE is 2.8%, while in Table 6, the average RMSE value is 1.5, and the MAPE is 2.1%.

**Figure 6:**
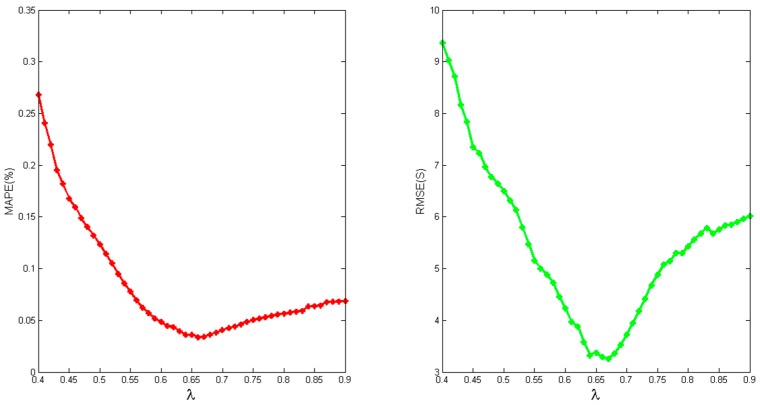
Accuracy with different value of λ.

We run experiments with λ values from 0.4 to 0.9 and the method for getting the value of λ has been given in Section 3. As shown in Figure 6, the best RMSE and MAPE accuracy are achieved when the λ value is at about 0.67. When λ is less than 0.67, data consisting of (progress, timestamp) pairs are considered to be generated in the following phase, and few data would be used for finding the reasonable parameters; when λ is over 0.67, a similar situation happens as in Figure 4 and Figure 5.

Table 7 depicts differences between actual values and prediction values using Two-Phase Regression for prediction. The average values of MRSE and MAPE are 4.9 and 9.8%, respectively. A Sort sample is examined in Table 8, sharing a similar tendency as in Table 7. The Average RMSE value is 6.3, and the MAPE is 13.5%. Certain groups depict big differences between predicted and actual values, because the type of input data in those groups is rack-local, which means task allocation happens in the local rack where the node belongs. When rack-local files are replaced by data-local ones, through which task allocation only happens in the node, the average MRSE and MAPE for WordCount decrease to 3.6 and 5.5%, respectively, while for Sort, those values also reduce to 4.8 and 6.1%, respectively.

**Figure 7:**
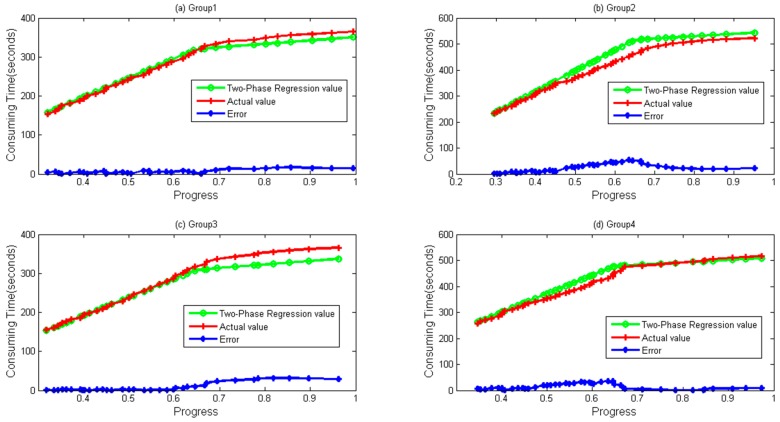
Two-Phase Regression values. Error obtained by Abs (Regression Value–Actual value). (**a**) Group 1; (**b**) Group 2; (**c**) Group 3; and (**d**) Group 4 data and their values calculated by Two-Phase Regression for WordCount sample.

**Figure 8:**
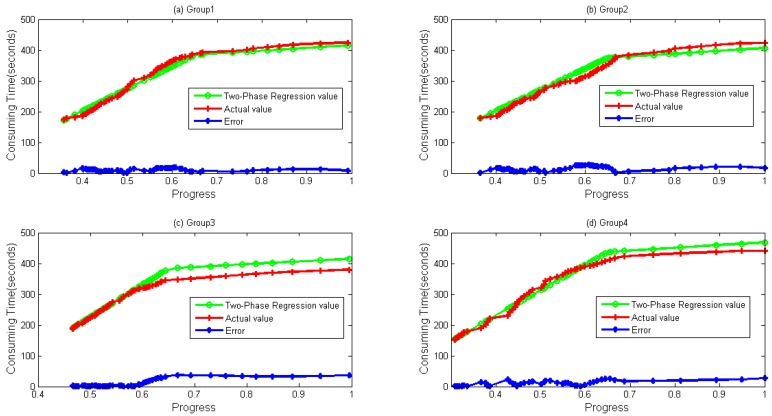
Two-Phase Regression values. Error obtained by Abs (Regression Value–Actual value). (**a**) Group 1; (**b**) Group 2; (**c**) Group 3; and (**d**) Group 4 data and their values calculated by Two-Phase Regression for Sort sample.

Four groups of data both for WordCount and Sort are depicted in Figure 7 and Figure 8, respectively. It can be seen during a whole lifetime, prediction values calculated by the TPR method fit the actual values well. Even when the progress is over 0.67, differences remain stable and near zero in most cases.

In Figure 5 and Figure 6, we can clearly find that when λ is close to 0.67, TPR has a higher accuracy. Exploring the procedure of the task, we find that local data combination happens from the stage, and those data are usually sorted on the same node. Based on the reason, the task speed is faster from the moment. In Figure 7 and Figure 8, it seems that bigger differences exist when a task is going to be accomplished. Actually, this is mainly due to the fact that some data need to be written to the distributed file system, and the speed of writing is easily influenced by the system load.

## 5. Conclusions

In this paper, detailed historical task execution data are collected by modifying MapReduce. A two-phase regression method has been presented for better prediction of the execution time of running tasks. According to the results, MAPE of running time prediction is on average 5.5% for WordCount sample and 6.1% for Sort sample. Periodical jobs have shown better accuracy according to the experimental results. In the future, we will study the relationship between starting speculation and load balancing. According to previous work, speculation would damage load balancing [40], and we will try to reduce the influence of starting the speculative execution strategy.

## Figures and Tables

**Table 1:** The detailed information of each virtual machine.

Node ID	Memory (GB)	Core Processors
Node 1	10	8
Node 2	8	4
Node 3	8	1
Node 4	8	8
Node 5	4	8
Node 6	4	4
Node 7	18	4
Node 8	12	8

**Table 2:** The detailed volume of input data and collected data.

Application	Input Data(GB)	Data Volume Collected (Groups)
Sort	30	326
WordCount	50	727

**Table 3:** Data of Map Tasks Collected by Modified Hadoop.

File Name	Size
attempt_1460032292591_0001_m_000000_0_ MAP	588 B
attempt_1460032292591_0002_m_000000_0_ MAP	903 B
attempt_1460032292591_0003_m_000000_0_ MAP	1.46 KB
attempt_1460032292591_0004_m_000000_0_ MAP	840 B
attempt_1460032292591_0005_m_000000_0_ MAP	1.23 KB
attempt_1460032292591_0006_m_000000_0_ MAP	1.33 KB
attempt_1460032292591_0007_m_000000_0_ MAP	861 B
attempt_1460032292591_0008_m_000000_0_ MAP	630 B
…	…

**Table 4:** Data Structure.

Progress	Timestamp
0.068	1460095563715
0.112	1460095567625
0.202	1460095587516
0.231	1460095607664
0.240	1460095611976
0.249	1460095633288
0.265	1460095633289
0.292	1460095633295
0.305	1460094649633
…	…

**Table 5:** Error evaluation during training phase for WordCount sample.

	**Group 1**	**Group 2**	**Group 3**	**Group 4**	**Group 5**	**Group 6**	**Group 7**	**Group 8**	
RMSE (S)	1.2	1.6	2.8	1.5	1.6	1.4	1.3	1.4	
MAPE (%)	1.7	2.8	2.8	1.7	2.4	2.7	1.6	3.1	
	**Group 9**	**Group 10**	**Group 11**	**Group 12**	**Group 13**	**Group 14**	**Group 15**	**Group 16**	
RMSE (S)	2.4	1.4	1.0	1.9	1.7	1.7	1.3	1.2	
MAPE (%)	3.3	2.4	1.1	5.3	5.5	2.4	1.7	2.0	
	**Group 17**	**Group 18**	**Group 19**	**Group 20**	**Group 21**	**Group 22**	**Group 23**	**Group 24**	**Average Value**
RMSE (S)	0.9	1.9	1.2	1.5	2.1	1.4	1.1	1.3	1.7
MAPE (%)	1.7	3.8	1.3	2.2	4.9	3.0	2.0	2.4	2.8

**Table 6:** Error evaluation during training phase for Sort sample.

	**Group 1**	**Group 2**	**Group 3**	**Group 4**	**Group 5**	**Group 6**	**Group 7**	**Group 8**	
RMSE (S)	1.6	1.5	1.4	1.1	1.6	2.4	1.7	1.5	
MAPE (%)	1.4	1.2	1.0	0.8	1.3	7.3	1.8	1.4	
	**Group 9**	**Group 10**	**Group 11**	**Group 12**	**Group 13**	**Group 14**	**Group 15**	**Group 16**	
RMSE (S)	1.6	1.6	3.1	1.6	2.4	2.4	0.9	2.1	
MAPE (%)	1.5	2	8	1.7	3.9	7.9	1.8	2.7	
	**Group 17**	**Group 18**	**Group 19**	**Group 20**	**Group 21**	**Group 22**	**Group 23**	**Group 24**	**Average Value**
RMSE (S)	1.7	0.6	2.3	1.6	1.3	1.8	1.4	1.6	1.5
MAPE (%)	1.8	6.8	4.6	1.5	0.9	2.8	1.5	2.1	2.7

**Table 7:** Error evaluation in predicting phase for WordCount sample.

	**Group 1**	**Group 2**	**Group 3**	**Group 4**	**Group 5**	**Group 6**	**Group 7**	**Group 8**		
RMSE (S)	1.8	3.5	5.7	2.7	3.6	2.8	3.5	1.9		
MAPE (%)	1.7	5.6	4.9	3.3	5.6	3.5	5.1	2.4		
	**Group 9**	**Group 10**	**Group 11**	**Group 12**	**Group 13**	**Group 14**	**Group 15**	**Group 16**		
RMSE (S)	10.9	6.7	3.7	5.2	2.7	4.4	3.1	1.9		
MAPE (%)	21	17.2	7.4	10.2	4.7	6.2	4.3	2.2		
	**Group 17**	**Group 18**	**Group 19**	**Group 20**	**Group 21**	**Group 22**	**Group 23**	**Group 24**	**Average Value**	**Corrected Value**
RMSE (S)	3.1	7.4	3.2	10.0	11.4	7.4	9.4	1.8	4.9	3.6
MAPE (%)	6.0	18.5	4.0	18.9	33.5	20.8	26.5	1.5	9.8	5.5

**Table 8:** Error evaluation in predicting phase for Sort sample.

	**Group 1**	**Group 2**	**Group 3**	**Group 4**	**Group 5**	**Group 6**	**Group 7**	**Group 8**		
RMSE (S)	3.4	4.4	4.2	4.1	2.8	9.4	3.4	4.1		
MAPE (%)	2.7	3.7	4.5	5.4	2	78.0	3.6	4.9		
	**Group 9**	**Group 10**	**Group 11**	**Group 12**	**Group 13**	**Group 14**	**Group 15**	**Group 16**		
RMSE (S)	4.3	3.7	9.3	7.8	10.9	3.7	13.6	5.0		
MAPE (%)	3.4	3.3	40.3	12	28.0	7.7	12.3	10.3		
	**Group 17**	**Group 18**	**Group 19**	**Group 20**	**Group 21**	**Group 22**	**Group 23**	**Group 24**	**Average Value**	**Corrected Value**
RMSE (S)	3.8	7.6	10.4	3.7	9.4	6.7	10.8	3.7	6.3	4.8
MAPE (%)	3.5	17.3	25.4	2.8	11	11.2	25.8	4.2	13.5	6.1
